# High-risk disease in newly diagnosed multiple myeloma: beyond the R-ISS and IMWG definitions

**DOI:** 10.1038/s41408-022-00679-5

**Published:** 2022-05-30

**Authors:** Patrick Hagen, Jiwang Zhang, Kevin Barton

**Affiliations:** 1 Department of Hematology/Oncology, Loyola University Medical Center, Maywood, IL 60153 USA; 2 Department of Pathology and Department of Radiation Oncology, Loyola University Medical Center, Maywood, IL 60153 USA; 3 Department of Cancer Biology, Oncology Institute, Cardinal Bernardin Cancer Center, Loyola University Medical Center, Maywood, IL 60153 USA

**Keywords:** Myeloma, Genetic translocation, Risk factors, Cancer genetics

## Abstract

Multiple myeloma (MM) is an acquired malignant plasma cell disorder that develops late in life. Although progression free and overall survival has improved across all age, race, and ethnic groups, a subset of patients have suboptimal outcomes and are labeled as having high risk disease. A uniform approach to risk in NDMM remains elusive despite several validated risk stratification systems in clinical use. While we attempt to capture risk at diagnosis, the reality is that many important prognostic characteristics remain ill-defined as some patients relapse early who were defined as low risk based on their genomic profile at diagnosis. It is critical to establish a definition of high risk disease in order to move towards risk-adapted treatment approaches. Defining risk at diagnosis is important to both effectively design future clinical trials and guide which clinical data is needed in routine practice. The goal of this review paper is to summarize and compare the various established risk stratification systems, go beyond the R-ISS and international myeloma working group risk stratifications to evaluate specific molecular and cytogenetic abnormalities and how they impact prognosis independently. In addition, we explore the wealth of new genomic information from recent whole genome/exome sequencing as well as gene expression data and review known clinical factors affecting outcome such as disease burden and early relapse as well as patient related factors such as race. Finally, we provide an outlook on developing a new high risk model system and how we might make sense of co-occurrences, oncogenic dependencies, and mutually exclusive mutations.

## Introduction

With the advent of new therapeutics and the increasing utilization of high-dose melphalan and autologous stem cell transplantation (ASCT) over the last 20 years, 5- and 10-year overall survival (OS) have improved across all age, race, and ethnic groups in multiple myeloma (MM) [1]. These benefits are more tempered in those with high-risk disease with revised international staging system (R-ISS) stage III patients achieving only a 24% 5-year progression-free survival (PFS) and 40% 5-year OS [2]. It is critical to identify high-risk patients at diagnosis in order to move away from treatment adapted to patient's physiological/chronological age and comorbidities and rather toward the establishment of risk-adapted treatment approaches.

A uniform approach to risk in NDMM remains elusive despite several validated risk-stratification systems in routine clinical use. This is a direct consequence of our rapidly expanding ability to evaluate genomic level data as well as an ever-expanding amount of patient-level clinical data. The accurate assessment of risk at diagnosis is important for many reasons including but not limited to:

1. The longest remission period is achieved by initial therapy and thus the duration of the first remission is one of the most important factors impacting patient prognosis
2. Accurate definition of risk for clinical trial enrollment
3. Establishing which clinical data should be obtained routinely in practice to define risk.

There is significant heterogeneity in the various risk-stratification systems currently utilized as outlined in Table 1. While we attempt to capture risk at diagnosis, the reality is that many important prognostic characteristics remain ill-defined as some patients relapse early who were defined as low risk based on their genomic profile at diagnosis. The goal of this review paper is to summarize and compare the various established risk-stratification systems and go beyond the R-ISS and international myeloma working group (IMWG) risk stratifications to evaluate specific molecular and cytogenetic abnormalities and how they impact prognosis independently. We explore the wealth of new genomic information from recent whole-genome/-exome sequencing as well as gene-expression profile data and review known clinical factors impacting outcome such as disease burden and early relapse as well as patient-related factors. Finally, we provide an outlook on developing a new high-risk model system and how we might make sense of co-occurrences, oncogenic dependencies, and mutually exclusive mutations.

**Table 1:** Different methodologies of risk stratification.

	Serum features	Genomic features	Proposed clinical definition of high risk:	% defined as high risk	Definition of high risk	Outcomes based on risk	Additional important notes
ISS [3]	Serum β2-microglobulin Serum albumin	None	NA^a^	33.6%	ISS stage III: Serum β2-microglobulin >5.5 mg/L	Median OS (months) • Stage I: 62 • Stage II: 45 • Stage III: 29	B2-microglobulin: indicative of increased tumor burden and declining renal function Serum albumin: driven by inflammatory cytokines such as IL-6 and the bone marrow microenvironment
R-ISS [2]	LDH Serum β2-microglobulin Serum albumin	del(17p)^b^ t(4;14) t(14;16)	NA^c^	10%	ISS stage III and either high-risk CA by iFISH or high LDH	5-year OS: • Stage 1: 82% • Stage 2: 62% • Stage 3: 40%	Stage 3 patients have a median PFS of 29 months and median OS of 37 months [54]
IMWG [5]	Serum β2-microglobulin Serum albumin	del(17p)^b^ t(4;14) +1q21	Median OS <2 years	20%	ISS II/III and t(4;14) or 17p13 del by iFISH	Median OS: • Low risk: >10 years • Standard risk: 7 years • High risk: 2 years	High-risk group with a 4-year PFS of 12% and OS of just 35% Low-risk group consists of ISS I/II and absence of t(4;14), 17p13 del or +1q21 and age <55 years
mSMART [55]	LDH Serum β2-microglobulin Serum albumin	Ploidy status t(4;14) t(14;16) t(14;20) t(11;14) t(6/14) del(17p) and p53 deletion deletion 13 gain 1q GEP	NA^d^	20%	High-risk genetic Abnormalities • t(14;16); t(14;20); • Del17p or p53 mutation GEP: high-risk signature	Median OS: • High risk: 3 years • Intermediate risk: 4–5 years • Standard risk: 8–10 years	• Trisomies may ameliorate high-risk genetic abnormalities • High plasma cell S-phase also defines high risk: cutoffs vary • Standard risk includes all others including trisomies, t(11;14), and t(6;14) • t(4;14): re-classified as intermediate risk
EMC92/SYK92 –MMprofiler [30]	None	High-risk survival signature of 92 genes^e^	Median OS <2 years	18–20%	Two-tiered system of high and standard risk	Reduced OS with HR of 2.06 to 5.23 in validation cohorts amongst the TT2, TT3, APEX, and MRC-IX studies	In multivariate analyses, the signature was proven to be independent of the currently used prognostic factors
UAMS GEP70 or MyPRS [28]	None	High-risk survival signature of 70 genes^e^	"early disease-related death"	13–14%	Two-tiered system of high and standard risk	HR for high v standard-risk GEP: • EFS: 3.41 (*P* = 0.002) • OS: 4.75 (*P*<0.001)	Standard-risk patients with a 5-year continuous complete remission of 60% vs. 3-year rate of only 20% in those with a high-risk "Early disease-related death" definition not clear in the primary literature
CoMMpass [19]	LDH	^f^TP53 mutation λ-chain translocation IGLL5 mutation	Time to progression (TTP) of < 18 months	20.6%	TTP < 18 months: high-risk TTP >18 months: low risk	Median OS in months: • High risk: 32.8 • ISS III: 54 • Baseline high-risk CA: 65	TTP 18-month cutoff chosen because time to ASCT was ~6 months and many MM studies define early PD as relapse within 12 months from ASCT
Myeloma Genome Project [6, 17]	Serum β2-microglobulin Serum albumin	TP53 inactivation +1q amp	NA^g^	6.1%	Biallelic TP53 inactivation or amp of CKS1B (1q21) on the background of ISS stage III	High risk: • Median PFS: 15.4 months • Median OS: 20.7 months	1q amplification considered ≥ 4 copies LDH values were not universally available preventing the calculation of R-ISS thus ISS and IMWG risk criteria were used
Cytogenetics Prognostic Index [9]	None	del(17p) t(4;14) del(1p32) 1q21 gain trisomies 3, 5, and 21	NA	11–18%	Prognostic Index >1 defined high risk^h^	5-year survival: • High risk: <50% • Int risk: 50–75% • Low risk: >75%	The main objective was to develop and validate a prognostic model based on the seven cytogenetic abnormalities

*amp* amplification, *ASCT* autologous stem cell transplantation, *CA* cytogenetic abnormalities, *GEP* gene-expression profile, *HR* hazard ratio, *iFISH* interphase fluorescent in situ hybridization, *ISS* International Staging System, *IMWG* International Myeloma Working Group, *Int* intermediate, *LDH* lactate dehydrogenase, *MM* multiple myeloma, *NA* not applicable/not available, *OS* overall survival, *PD* progressive disease, *PFS* progression-free survival, *R-ISS* Revised International Staging System, *TTP* time to progression.

^a^Univariate and multivariate analyses were used to explore three modeling approaches with the most significant prognostic factors assessed using the following methods: (1) the weighted variable model; (2) the model based on the number of risk factors occurring in an individual patient; and (3) the survival tree model in which risk factors present at each branch point are sequentially reassessed.

^b^iFISH: early studies showed the power of MM-specific abnormalities on metaphase cytogenetics and their association with inferior survival. This assay relies on the presence of actively dividing cells, and as terminally differentiated B cells, plasma cells have limited proliferative capacity [38]. Consequently, only one-third of MM patients have metaphase cytogenetic abnormalities at diagnosis. Interphase FISH is a more sensitive modality for identifying specific cytogenetic abnormalities associated with inferior survival and is used to depict risk for both the R-ISS and IMWG staging systems.

^c^Utilized different statistical models to best partition risk. K-adaptive partitioning dedicated to censored survival data (minimax-based partitioning rule by log-rank test) was used for ISS/CA/LDH grouping; this routine gave an optimal number of three subgroups (R-ISS I, II, and III).

^d^Partitioned into three groups based on data from multiple centers.

^e^See gene-expression profile section for further details and references.

^f^Factors associated in multivariate analyses with time to progression of less than 18 months.

^g^Utilized different statistical models to best partition risk, please refer to Walker et al. [17].

^h^Refer to Perrot et al. [9] for more information on how the prognostic index was calculated.

## General risk-stratification systems

The international staging system (ISS) is one of the earliest validated risk stratification for NDMM patients [3]. The ISS is a biological staging system predicting risk based on rising serum β_2_-microglobulin (β2M) and falling serum albumin. Subsequent to the ISS development, chromosomal abnormalities (CA) detected by interphase fluorescent in situ hybridization (iFISH) have become a standard of care in risk stratifying MM patients. Certain high-risk changes including del(17p), translocation t(4;14), and translocation t(14;16) have been established [4]. In 2014, the IMWG published an updated risk stratification focusing on differentiating high from standard-risk patients combining the ISS with certain high-risk iFISH changes including t(4;14), del17p13, and +1q21 [5].

The R-ISS combines iFISH changes, serum lactate dehydrogenase (LDH), and ISS features and is the most widely recognized risk-stratification tool for NDMM patients [2]. The R-ISS is a simple but clinically useful system predictive of both OS and PFS in NDMM. Although it incorporates important genomic markers including t(4;14), t(14;16), and del17p, it does not include 1q gain/amplification, an increasingly important prognostic marker [6], or mutational data from TP53. Importantly, in order to be R-ISS stage III, patients must also be ISS III with the biological marker β2M elevated to ≥ 5.5 mg/L. A significant portion of patients will be R-ISS stage I or II despite having high-risk iFISH changes. In a recent report by Corre et al. [7] evaluating del(17p) and TP53 mutations in NDMM patients, 73% of the patients with del(17p) alone and 52% of those with TP53 biallelic inactivation were not International Staging System (ISS)−3 and thus not classified in the R-ISS 3 subgroup. Further, β2M may indeed be a biological maker of high-risk disease but likely the inherent high-risk genomic features drive this as Bolli et al. found that 1q amplification correlated with higher β2M [8]. Finally, neither the R-ISS nor the IMWG weight cytogenetic findings. A recent report by the Intergrouped francophone Du Myelome (IFM) has shown that a weighted cytogenetic risk stratification based on certain high-risk lesions such as a del(17p), del(1p32), gain 1q, t(4;14), and trisomy 21 may have the ability to more accurately risk-stratify patients [9]. Unfortunately, the vast majority of patients included in this study were not treated with modern induction regimens. This brings up a frequent challenge when evaluating prognostic scores in NDMM given the quickly evolving treatment landscape and lack of treatment adjustment into currently utilized risk-stratification systems.

## Beyond the R-ISS: molecular subgroups and cytogenetic abnormalities

In addition to traditional staging systems, there are well-established high-risk features in MM that portend to poor outcomes. These features include other molecular subgroups (primarily translocations into the immunoglobulin heavy-chain locus and copy number abnormalities (CNAs)) as well as new and emerging structural, mutational, and copy number drivers based on next generational sequencing. MM may have chromosomal aberrations carried by only a subset of tumor cells, and the cytogenetic heterogeneity of individual cases reflects the coexistence of cytogenetically defined aberrant plasma cell clones. A surrogate marker of clone size may include the percentage of cells harboring specific cytogenetic abnormalities detected by FISH. Although the European Myeloma Network (EMN) has recommended relatively conservative cutoff values of 10% for fusion or break apart probes and 20% for numerical abnormalities (similar cutoffs were utilized for the R-ISS staging system), so far no uniform cutoffs have been applied, and the cutoffs used in different centers are inconsistent.

## Well-established molecular subgroups: translocations into the immunoglobulin heavy-chain locus

Most translocations into the immunoglobulin heavy-chain locus located at 14q32 are seen in greater than 40% of NDMM patients [4, 6]. The IgH locus at 14q32 is transcriptionally active in B cells, and the translocation of putative oncogenes to this region and their subsequent dysregulated expression is considered a seminal event in the pathogenesis of most B-cell malignancies, including MM [10]. There are several known translocations of 14q32 with nonrandom partners, including the more commonly observed t(4;14) and t(11;14) translocations (30% of patients with MM) and the less common (⩽5% of patients) t(14;16), t(6;14), t(8;14), and t(14;20) translocations [10]. Each translocation subgroup is associated with deregulation of a D group cyclin either directly, such as occurs with the t(11;14) (cyclin D1) and t(6;14) (cyclin D3), or indirectly such as occurs with the t(4;14) or in the MAF translocation group which includes t(14;20) and t(14;16) [11]. These translocations ultimately lead to upregulation of oncogenes—including D-type cyclins (cyclin D1, D2 and D3), MAF family members (MafA, MafB, and c-Maf), c-MYC, the myeloma SET domain protein (MMSET), and the fibroblast growth factor receptor 3 (FGFR3)—and have been shown to influence patient prognosis.

### Adverse

The MAF translocation group includes the t(14;16) and t(14;20), both of which are rare in MM, but are thought to be associated with poor prognosis. The mechanism of this poor outcome is thought to involve the consequences of MAF upregulation, which include upregulation of cyclin D2, and its effects on cell interaction and upregulation of apoptosis resistance [11]. t(4;14) translocation leads to mutation of the MMSET gene that is known to have histone methyltransferase activity and is deregulated early on in the genesis of developing MM [12]. t(8;14) and MYC aberrations/translocations lead to upregulation of the MYC oncogene. The prevalence, pathogenesis, and supporting literature for both 14q32 translocations and CNAs dictating risk varies and is outlined in Table 2.

**Table 2:** High-risk molecular and cytogenetic subgroups; balanced translocations into the immunoglobulin heavy-chain locus and copy number abnormalities.

	Prevalence	Pathogenesis	High risk per R-ISS, IMWG, mSMART:	Literature addressing prognosis:	Notes
t(14;20)^a^	1–2%	MAF upregulation including upregulation of cyclin D2, effects on cell interaction and upregulation of apoptosis resistance	mSMART	Inconsistent due to rarity • Ross et al.: median OS 14.4 months [11] • Jurczyszyn et al.^b^: median PFS 30 months [56] • Shah et al. [57]: HR for OS of 1.90 (*P* = 0.0089) with only del(17p) showing worse prognosis	No large databases, cohorts of less than 50 patients Frequently found with other high-risk cytogenetic abnormalities: del(17p), t(4;14), t(14;16), del(13q), non-hyperdiploid karyotype
t(14;16)	1–2%	MAF upregulation including upregulation of cyclin D2 Effects on cell interaction and upregulation of apoptosis resistance	R-ISS mSMART	Inconsistent due to rarity: • Jurczysyn et al.^c^: median PFS 31 months; 5-year OS 55% [56] • Mayo Clinic and Medical Research Council group [58] showing poor outcomes • IFM studies showed neutral outcomes [59]	Data from Jurczysyn et al. do not vary significantly from R-ISS stage III outcomes. Of note, the R-ISS did not report specifically on outcomes of t(14;16) patients
t(4;14)	15%	MMSET on der(4) MMSET is known to have histone methyltransferase activity and is deregulated early on in the genesis of developing MM^d^ [9]	IMWG R-ISS mSMART	• Chan et al.: PFS of 33.5 months for 75 patients [60] • Mayo group has demonstrated improved median OS ~ 4–5 years [55] • Bolli et al.: On MV analyses, t(4;14) predicted both PFS and OS independently of other CAs [8]	• Compared to standard-risk patients who achieve median OS ~10 years, t(4;14) patients remain bad actors • Some frequently associated chromosomal changes worsen t(4;14) such as +1q, 1p32 and potentially 13q deletions
t(8;14) and MYC translocations	1–2%	Upregulation of the oncogene MYC^e^	none	• Myeloma IX trial: those with a MYC translocation had inferior PFS and OS on MV analyses [61] • CoMMpass study [22]: Only IgL-MYC translocations had worse PFS and OS^f^	Significant associations between Myc and other abnormalities highlight oncogenic dependencies Myc rearrangements can lead to deregulation of FAM46C which has been associated with hyperdiploid MM [17]
t(11;14)	15–20%	Upregulation of CCND1 [62] expression^g^ Express higher ratios of BCL2 (anti-apoptotic) to MCL1 (proapoptotic)	None	• Traditionally favorable/standard risk • Recent reports show t(11;14) is likely at best standard risk [63] • Connect MM registry data shows neutral risk but possibly increased risk in AA patients [64] • Mutations in CCND1 are key with poor survival amongst mutated v non-mutated t(11;14) patients: median OS 20.2 months vs. NR (*P* = 0.005) in the Myeloma XI trial [65]	t(11;14) associated with a characteristic lymphoplasmacytic morphology, light chain MM, rarer variants of MM (IgD, IgM, and nonsecretory), and expression of CD20 on the surface of PCs [66]
t(14;x)^h^	15–20%	Unknown	None	• Mao et al. [67]: t(14;x) lead to improved OS on MV analyses (HR = 0.51, 95% CI 0.30–0.85) • Kaufman et al. [68]: t(14;x) median PFS 26.6 and OS 92.8 months, not significantly different from the comparison general cohort	Despite its remarkable prevalence with t(14;unknown) being as common as t(4;14) or t(11;14), its impact on risk and prognosis is not well described albeit it is thought to be neutral
+1q	Overall: ~33% Gain: ~21.9% Amp: ~6.3%	Amplified CKS1B results in greater degradation of p27, activation of the Cdk/cyclin complex, and cell cycle upregulation by promoting the G1/S transition [13]	None	• Giri et al. [69]: 3578 NDMM patients, any chromosome 1 abnormality inferior OS (median OS 46.6 vs. 70.1 months) • Shah et al. [57]: UK Myeloma XI and IX trials, 1q gain inferior OS (HR 1.67; *P* = 3.30 × 10–5); amp 1q worse (HR 2.28; *P* = 2.32 × 10− 6) • MGP: both gain and amp of CKS1B associated with decreased PFS and OS [6] but amp worse • Gain and amp have been shown to impact OS in other cohorts [8, 14, 19, 26, 57]	Cutoff for a positive test remains controversial with the EMN 20% definition frequently employed but higher CCF may impact outcomes. An et al. showed that amongst patients with 1q21 gains a 20% CCF predicted PFS and OS but stratifying by increasing CCF had no impact on outcomes and likely at 20% CCF cutoff remains appropriate [70]
1p-	~10%	Deletion of CDKN2C, a tumor suppressor gene, leads to deregulation of the G1/S transition FAM46C promotes MM cell growth-inhibiting apoptosis	None	• Myeloma IX trial: inferior OS for both CDKN2C mutation at 1p32.3 as well as FAM46C at 1p12 [71] • IFM collection: In MV analyses of 1195 patients, 1p22 and 1p32 deletions both showed inferior OS [72]	Amplification of CKS1B is frequently associated with the deletion of the CDKN2C gene at the chromosome 1p32.3 (1p-) locus
del 13q/-13	del(13q): ~5% Monsomy 13: 35%	FISH probes to both putative tumor suppressor gene Rb-1 and to D13S319, a gene locus distal to Rb-1, showed inferior OS in NDMM patients. Exact mechanism though is unclear.	None	• Early studies showed inferior OS in NDMM patients but this may be due to co-occurring high-risk CAs [73] • In 1181 NDMM patients, on MV analyses monosomy 13 lead to worse OS with a HR of 1.27 (*P* = 0.022) while del(13q) with a HR of 0.48 (*P* = 0.006) [74]	• Deletions and abnormalities involving chromosome 13 were one of the earliest recognized high-risk features in NDMM [75] • Up to 90% of patients with t(4;14) have deletion 13q [76] • CCF has not been clearly defined, at CCF >25% it is likely co-occurring CAs, particularly t(4;14) and del(17p), drive poor clinical outcomes [70]
del17p	5–10%	Tumor suppressor gene but the exact mechanism by which del17p promotes aggressive disease biology remains unclear	R-ISS IMWG mSMART	Extensive data, see the manuscript as well as Table 3 [26]	TP53 induces clonal immortalization and survival of tumor cells as well as drug resistance which is thought to drive poor prognosis [77, 78]

*amp* amplification, ≥4 copies, *AA* African American, *CA* cytogenetic abnormalities, *CCF* cancer clone fraction, *EMN* European myeloma network, *HR* hazard ratio, *IFM* Institut Francophone du Mye´lome, *IMWG* international myeloma working group, *MGP* myeloma genome project, *MV* multivariate, *NDMM* newly diagnosed multiple myeloma, *NR* not reached, *OS* overall survival, *PCs* plasma cells, *PFS* progression-free survival, *Rb-1* retinoblastoma gene-1, *R-ISS* revised international staging system.

^a^While t(4;14) and translocation t(14;16) are included as high-risk chromosomal abnormalities in the R-ISS, other chromosome 14 translocations including t(14;20) are not but have been shown to be unfavorable.

^b^Five clinical centers in Germany, Italy, and the United States.

^c^In total, 213 patients with t(14;16) from 24 clinical centers in Germany, Italy, Spain, Israel, Poland, Romania, Czech Republic and the United States.

^d^The karyotypically silent t(4;14) translocation, undetectable by conventional cytogenetic analysis, was identified first based on breakpoints on chromosome 4 in the FGFR3 gene and subsequently involving the MMSET gene (MMSET: multiple myeloma SET domain; also known as Wolf-Hirschhorn syndrome candidate 1 (WHSC1) or nuclear receptor-binding SET domain 2 (NSD2)). The t(4:14) translocation was the first example of an IgH translocation that simultaneously dysregulated two genes with oncogenic potential: FGFR3 on der(14) and MMSET on der(4). Importantly, FGFR3 shows only weak transforming activity and is eventually lost in 30% of patients suggesting that it is not the main oncogenic factor [76], whereas MMSET is known to have histone methyltransferase activity and is deregulated early on in the genesis of developing myeloma [12].

^e^Translocations at 8q24 have been shown to portend to poor outcomes and 8q24 breakpoints have been found to partner with immunoglobulin enhancers (IGH, IGK, and IGL), important B-cell maturation loci including (XBP1, FAM46C, CCND1, KRAS) and other superenhancers, such as NSMCE2, TXNDC5, FOXO3, IGJ, and PRDM1 [22, 61].

^f^These data indicate that aberrant MYC expression resulting from MYC amplification or translocation is a common feature of myeloma, but the IgL-MYC translocated subset is unique among MYC alterations in that it portends a very poor prognosis. On the CoMMpass study, patients with an IgL translocation did not benefit from IMiD-containing therapies that target the lymphocyte-specific transcription factor Ikaros which is bound at high levels to the IgL enhancer. Also, 78% of IgL-MYC translocations co-occur with hyperdiploid disease, a marker of standard risk, suggesting that IgL-MYC-translocated myeloma is being misclassified.

^g^Normal B-cells express cyclin D2 and D3 [62].

^h^t(14;x) The partner genes translocated with the IgH vary in their impact on risk and prognosis in NDMM patients. Not infrequently the IgH spilt can be detected by FISH but no specific partner chromosomes can further be identified [68].

## Well-established molecular subgroups: copy number abnormalities

Additional copy number gains and losses occur frequently with the most frequent being del 13q (59%), +1q (40%), del14q (39%), del6q (33%), del1p (30%), and del17p (8%). Table 2 outlines key features of CNAs with special attention below to 1q gain/amplification and del(17p) as these likely represent the most deleterious genomic changes in NDMM.

### 1q amplification (v gain)

The gain/amplification of CKS1B gene at chromosome region 1q21 (1q+) is one of the most common secondary genetic abnormalities in MM and is seen in about one-third of NDMM patients [7]. CKS1B is an essential protein for cell growth and division and is a member of the cyclin kinase subunit 1 protein family. It is expressed universally in the bone marrow and associates with p27kip1-Cdk/cyclin complex and acts as a cofactor for Skp2-dependent ubiquitination of p27 [13]. An amplified CKS1B results in greater degradation of p27, activation of the Cdk/cyclin complex, and cell cycle upregulation by promoting the G1/S transition and plays a critical role in cell cycle progression and MM cell survival.

Various 1q states are seen in NDMM patients including diploid, gain of 1q (three copies of 1q), and amplification of 1q (≥4 copies of 1q). The differential impact on prognosis between gain and amplification remains to be completely elucidated but any additional copies of 1q has been shown to lead to inferior outcomes. The impact of copy number on long-term outcomes is variable but ≥4 copies or amplification typically drives the most dismal PFS and OS [6]. While many postulate that del(17p)/TP53 mutation is the most impactful driver of prognosis, the recently updated data on 1q amplification from the FORTE trial calls this into question where an intensified treatment approach improved outcomes in all groups save those with 1q amplification [14].

### del(17p)

Cytogenetic analysis of chromosome 17p deletions which spans the TP53 gene is typically performed by iFISH probes against 17p and does not probe TP53 in isolation. Although the clinical relevance of del17p is well established in MM, the exact mechanism by which del17p promotes aggressive disease biology remains unclear. As in other tumor types, TP53 mutations in MM are spread across the entire gene, with many mutations occurring within the DNA-binding domain [15]. The length of the deleted region can vary from a few megabases (MBs) to deletion of the entire short arm of chromosome 17. The TP53 gene is located in the minimally deleted region (0.25 MB) suggesting that it is a critical gene in the 17p13 region. However, a deletion event usually involves several genes and co-deletion of TP53 along with Eif5a and Alox15b has resulted in more aggressive disease [15]. It remains unclear how genes other than TP53 contribute to tumorigenesis. Missense mutations of TP53 might associate with even worse outcome in some cases as they produce mutant TP53 proteins that not only result in loss of normal TP53 function but also gain of oncogenic functions [16]. From the myeloma genome project (MGP), Walker et al. demonstrated that TP53 deletion is the most common abnormality at 8%, followed by mutation (~6%) and biallelic inactivation (~4%). Of note, TP53 mutation has been identified as a driver mutation in MM and is one of the few driver mutations with prognostic power [17].

Early studies suggested an association between deletion on one allele and mutation on the second allele putatively resulting in complete inactivation of P53 function [18]. The relationship between mono and biallelic del(17p) and TP53 mutational status remains to be clarified and Table 3 summarizes the known prognosis of biallelic vs. haploinsufficiency. Further, what defines a positive test for del(17p) remains controversial with cancer clone fraction (CCF) positivity rates vary based on cutoffs. The known impact of CCF is also summarized in Table 3.

**Table 3:** del(17p) biallelic vs. haploinsufficiency and cancer clone fraction (CCF).

Reference and setting	Number of patients with del(17p)	Incidence del(17p)/TP53 mutation	CCF cutoff for del(17p) detection	% double hit^a^ among del(17p)	Impact of biallelic/double hit^a^ vs. monoallelic inactivation^b^	CCF impact on outcomes
Corre et al. [7]; NDMM treated with intensive strategy in France	121	NA	>55%	37.2%	TP53 biallelic inactivation vs. del(17p) alone: median OS 36.0 vs. 52.8 months (*P* = 0.004) del(17p) monoallelic v control (*n* = 2505): OS 52.8 vs. 152.2 months^c^	CCF >55% choosen based on Thakurta et al. [79] data that showed virtually all patients with TP53 biallelic inactivation display the deletion in more than 55% of plasma cells
Thakurta et al. [79]; MGP	108^d^	NA	NA	25.9%	Double-hit PFS (*P*<0.01) and OS (*P*<0.01) worse compared to haploinsufficiency	CCF <55% vs. >55%: • OS: 36 vs. 84.1 months • PFS: 14.3 vs. 23.9 months
Walker et al. [6, 26]; MGP	97^d^	9%^e^	NA	3.7%	On MV analyses: • PFS: only biallelic TP53 interactions significant • OS: only biallelic TP53 interactions significant	The CCF of the 63 driver genes higher in oncogenes than tumor suppressor genes (*P* = 0.001). TP53 was the notable exception, with a high CCF
D'Agostino et al. [19]; CoMMpass study (NCT01454297)	111	13%	20%	24%	Early PD^f^ • del(17p): 17.1% • TP53 mutation: 50%, • Biallelic: 41.4% MV analysis: TP53 mutation OR 3.78 (*P* < 0.01) predicts PD within 24 months	CCF >50% vs. 20% predictive of early progression or death within 24 months: 25% vs. 17.1%
Thanendrarajan et al. [80]; total 3–5 trials	76	10%	20%	9.2%	Homozygous del17p or both del17p and TP53 mutation as compared to halploinsufficiency • 3-year OS: 84% vs. 29% (*P* = 0.02) • 3-year PFS: 73% vs. 29% (*P* = 0.04)	CCF >60% optimal^g^ • HR for PFS: 1.53 (*P* = 0.03) • HR for OS: 1.69 (*P* = 0.013) CCF based on GEP70 score: • GEP70 low risk: no cutoff predictive • GEP70 high risk: CCF >60% predictive ◦ 3-year OS 73 vs. 87%; *P* = 0.002 ◦ 3-year PFS 64 vs. 81%; *P* = 0.004
Shah et al. [40]; UK NCRI Myeloma IX and XI trials	192	10.8%	10–20%	2.4%	^h^MV analyses OS Myeloma XI trial: biallelic v monoallelic deletion/loss of TP53: • Heterozygous TP53 deletion/loss: HR 2.13 (1.58–2.86)/HR 2.18 (1.35–3.52) • Homozygous TP53 deletion/loss: HR 2.98 (1.22–7.26)/HR 4.31 (2.03–9.18)	^i^Three groups associated with OS: • CCF 10–20%: HR of 1.8 (*P* = 0.01) • CCF >50%: HR of 2.9 (*P* = 5.6 X 10–7) • CCF 95–100%: HR of 2.2 (*P* = 0.0002)
Avet Loiseaeu et al. [81]: IFM99	58 del(17p) patients	11%	10%	NA	NA	CCF of 60% for del(17p): event-free survival 14.6 vs. 34.7 months
Merz et al. [82]; single center Germany	110 del(17p) patients	NA	10%	NA	NA	CCF cutoff >60% vs. 10–60%: • PFS: 19 vs. 26 months, *P* = 0.03 • OS: 30 vs. 54 months, *P* = 0.09
An et al. [70]; single center China	22 del(17p) patients	6.6%	20%	NA	NA	MV analyses: CCF>50% • PFS: HR, 2.455; *P*<0.001 • OS: HR, 1.754; *P*<0.001
Cohen et al. [83]; multi-institution (*n* = 8)	60 del(17p) patients	NA	5%	NA	NA	CCF cutoff >50%: • PFS: HR 1.7; *P* = 0.08 • OS: no CCF predictive
Lakshman et al.; single institution [84]	310	NA	7%	NA	NA	Median CCF of the entire cohort: 69.5% • PFS: 40% (*P* = 0.014) and 50% (*P*=0.027) CCF predicted PFS • OS: cutoff values from 20 to 60% did not predict OS

*CCF* cancer clone fraction, *CN* copy number, *HR* hazard ratio, *iFISH* interphase fluorescent in situ hybridization, *MGP* myeloma genome project, *MV* multivariate analyses, *NA* not applicable/not available, *NDMM* newly diagnosed multiple myeloma, *OS* overall survival, *OR* odds ratio, *PD* progressive disease, *PFS* progression-free survival.

^a^Double hit: displaying del(17p) and an additional TP53 mutation.

^b^Halploinsufficiency: either del17p alone or TP53 mutation alone.

^c^Haploinsufficiency still leads to poor outcomes.

^d^TP53 mutation based on whole-exome/genome sequencing as opposed to iFISH.

^e^In the full dataset, TP53 deletion was seen in 9.0% (97/1074) and mutations in 5.5% (70/1273) of patients. Any event at TP53 was found in 11.3% and biallelic events in 3.7% of patients. Importantly, when mutations of TP53 are taken into account, del(17p) was not prognostically important.

^f^Early progressive disease was defined as time to progression of less than 18 months.

^g^MV analyses: del17p always entered the final model whether the cut-point used was 20%, 40%, 60%, or 80%, suggesting that del17p is indeed an independent prognostic factor.

^h^Homozygous TP53 deletion was associated with a very short median OS of 22.4 months and an HR for OS of 3.7 (95% CI, 1.5–8.9; *P* = 0.004).

^i^TP53-deleted tumors were divided into three equal-sized subgroups based on MLPA (multiplex ligation-dependent probe amplification) values: deleted tumors (*n* = 67; MLPA 0.7–0.8—corresponding to a CCF of 10–20%); intermediate clonal tumors (*n* = 64; MLPA 0.55–0.7—corresponding to a CCF of greater than 50%); clonally TP53-deleted tumors (*n* = 61; MLPA <0.55—corresponding to a CCF of ~95–100%).

The use of different thresholds/CCFs, different size datasets, as well as different treatment regimens have resulted in discordance in the reported prognosis of del17p. Regardless, when detected del(17p) is ubiquitously adverse. The R-ISS, IMWG, and mSMART staging systems as well as whole-genome/-exome sequencing data from both the myeloma genome project [6] as well as the IMWG CoMMpass study [19] have all clearly shown dismal outcomes in del(17p) patients. When incorporating RNA alterations and gene-expression profiling it remains predictive of both PFS and OS as well.

### Hyperdiploid, tetraploid, and trisomies

Hypodiploid karyotypes or hyperhaploid karyotypes are associated with an adverse prognosis in NDMM. Tetraploidy is an independent marker associated with significantly shorter OS [20]. It is well described that several high-risk lesions frequently co-occur with standard-risk patients and that hyperdiploid myeloma (HD-MM), although generally agreed upon to be protective [21], is biological heterogeneous as exemplified by the fact that 78% of IgL-MYC translocations co-occur with HD-MM [22]. Further, among HD-MM, patients with trisomy 21 have poor outcomes [23] although this is controversial and being increasingly challenged.

### The challenge and applicability of traditional iFISH risk stratification

The IMWG consensus statement describes clinical iFISH as the standard approach for detecting CAs and the R-ISS staging system followed the same methodology. However, within the R-ISS inconsistencies existed in defining positive cytogenetic abnormalities and the cutoff levels were not identical ranging from 8 to 20% for numerical aberrations and from 10 to 15% for immunoglobulin heavy-chain translocations. Further, in routine clinical practice, more heterogeneity exists with some labs not performing the required purification or dual staining and as with the R-ISS data the detection limits and positivity thresholds vary between institutions. This heterogeneity may limit the utility of the R-ISS and IMWG staging systems particularly when applied after collaborating data from multiple institutions. More recently, extensive collections of MM genomic data are being utilized to further elucidate risk in NDMM patients but they too have not escaped this challenge. For example, the CoMMpass study (NCT01454297) has provided an unprecedented platform for genomics and outcomes research in MM but one of the few critiques stems from the heterogeneity in cytogenetic analysis. In an audit of the top ten recruiting sites, significant discordance was found between the local data extraction and their central audit with variability in the FISH probes utilized, number of cells counted, and sorting techniques [24]. Of note, traditional FISH studies are quite expensive further motivating the field to move beyond traditional FISH studies toward next-generation sequencing tools.

Seq-FISH with next-generation sequencing tests can be designed to simultaneously detect the copy number abnormalities and translocations detected by clinical FISH along with gene mutations that cannot. From the CoMMpass study, Goldsmith et al identified 672 patients with sufficient data to calculate R-ISS via Seq-FISH technique using calls on whole-genome sequencing (WGS) long-insert data with the threshold for a positive detection of a CNA by Seq-FISH being 20%. The R-ISS-NGS resulted in significant redistribution of patients from stage I into stage II. R-ISS-NGS stages II and III were associated with worse PFS and OS more so than the staging schema of the R-ISS [24]. Further, Miller et al. evaluated 339 patients also from the CoMMpass study and found Seq-FISH identified nearly all translocations as well as 30 translocations missed by clinical FISH [25]. Thus Seq-FISH has validated the prognostic power of the R-ISS and increased the sensitivity and reproducibility of identifying CAs. However, like gene-expression profiling described in detail below, the clinical application remains challenging given the laboratory experience and capabilities required as well as turnaround time in routine clinical practice.

### Making sense of co-occurrences, oncogenic dependencies, and mutually exclusive mutations

As more samples are sequenced in MM, co-occurrences or oncogenic dependencies between genomic markers are being increasingly described [6, 26]. This makes an exact assessment of the impact of specific cytogenetic abnormalities difficult especially when these abnormalities are considered in isolation and or when they are rare events such as t(8;14) or t(14;16). Prior to our ability to readily perform whole-genome sequencing, the number of known oncogenic dependencies were limited. However, large datasets such as the Myeloma Genome Project and the CoMMpass project have increased our awareness of co-occurring events. The co-segregation of these adverse prognostic factors emphasizes the need to adjust for potential confounding and should lead to improved risk stratification in NDMM patients. Further, understanding the biology of the tumors and how particular co-dependencies function and their potential reliance on similar pathways may lead to identifying new therapeutic targets.

## Whole-genome/-exome sequencing

Next-generation sequencing (NGS) technologies have allowed the identification of RNA transcript expression, genomic structural variants (translocations, deletions, insertions, inversions), single nucleotide variants, loss of heterozygosity, and copy number abnormalities affecting whole chromosomes, segments of chromosomes, and individual genes. Dozens of myeloma driver genes have been identified with the most common occurring in the RAS and NF-kB families [27]. Chromothripsis, a genomc event that leads to massive, clustered genomic rearrangements, is an emerging high-risk signature that is just recently being described. With newer technologies making whole-exome and whole-genome sequencing more readily available and less expensive, the ability to complete more comprehensive genomic profiling of MM patients is increasingly becoming a reality. This has renewed the importance of identifying and prognosticating driver mutations and additional genetic variants that might lead to improved patient expectations and ultimately therapeutic advancements.

The MGP, CoMMpass study, as well as work done by a collaboration of US and European centers published by Bolli et al. [8] has expanded our knowledge of the genomic environment in which MM develops and importantly identified novel risk factors leading to poor outcomes. Several conclusions can be safely made after reviewing this data including:

- del(17p)/TP53 mutations a well as +1q amplification are powerful drivers of poor prognosis
- Many novel driver and oncogenic genes remain to be explored
- Loss of heterozygosity [6, 17] (LOH) and an APOEBEC [6, 17] signature impact prognosis
- Burden of driver gene and overall somatic missense mutation drive poor outcomes
- Genomic clusters exist and dictate prognosis
- Certain genomic pairings leading to "double hit" genotypes dictate dismal outcomes.

Table 4 summarizes key findings in the most recently reported large patient datasets with whole-genome/-exome sequencing available.

**Table 4:** Whole-genome/-exome sequencing.

	Number of patients	Source of patients	Genomic findings of note	High-risk factors identified
Myeloma genome Project [6, 17]	1273	• Myeloma XI trial, • Dana-Faber Cancer Institute/Intergroupe Francophone du Myelome • Multiple Myeloma Research Foundation CoMMpass study	• 63 driver genes identified including novel oncogenes PTPN11 (activator of MEK/ERK signaling), PRKD2 (protein kinase D), IDH1, and IDH2 (DNA methylation), and SF3B1 (spliceosome factor) • Identified novel tumor suppressor genes including UBR5 (a ubiquitin ligase) and HUWE1 (a ubiquitin ligase that can affect MUC expression via MIZ1) • Extent of LOH was positively correlated with the APOBEC signature (*P* = 0.039), loss of TP53 (*P* = 0.001), and presence of mutations in at least 1 of 15 genes involved in homologous recombination deficiency (*P* < 0.001)	• Of 63 driver genes: only TP53, TRAF3, and TGDS had an impact on outcome • Driver gene mutational burden leads to worse PFS/OS • Two markers of genomic instability were associated with outcomes: APOBEC mutational signature and LOH. Other studies have also shown APOBEC mutational signatures to be high risk [64] • Identified 9 separate copy number groupings with prognostic value ◦ Cluster 7: gain of 1q, t(4;14) and t(14;16) • No correlation between ISS stage and distribution of genetic features • On multivariate modeling: ◦ t(4;14) and biallelic TP53 inactivation predictive of PFS ◦ biallelic TP53 and CKS1B amplification predictive of OS ◦ "Double-Hit": median PFS 15.4 and OS 20.7 months ▪ biallelic inactivation of TP53 or ▪ ISS III with amplification of CKS1B:
Bolli et al. [8]	418	• 373 MM patients at diagnosis • Added 45 patients from a previously published WES study	• Many genes showed an excess of variants of possible oncogenic potential but unknown pathogenesis • FAT1, FAT3, FAT4, DNAH9, DNAH11, PCLO • Sporadic oncogenic mutations with potential clinical impact in CRBN and IKZF1^a^ • Possible novel tumor suppressors XBP1 and PRMDA which control plasma cell development • Frequently alleles of driver genes were multiply mutated -up to 5 for TP53- at a subclonal level • CCF of mutations did not influence OS save a trend towards improved OS for TP53 • Double hit^b^ frequently noted for TP53, CYLD, and TRAF3 mutations	• "Double Hit": ◦ both t(4;14) and PRDM1: median OS of 265 days ◦ t(4;14) and TP53 mutations: median OS of 228 days • Clusters of patients stratified based on the overall number of mutations and number/type of CAs that lead to distinct effects on survival ◦ Clusters predict PFS/OS: Cluster 2 showing the worse median OS—1973 days ◦ Cluster 2 was enriched for IGH translocations, the highest number of CAs, was enriched for amp(1q), del (13), del(17p), deletions of BIRC2/3 and XBP1 and carried more TP53 mutations • The only mutated gene with a clear prognostic impact on both PFS and OS was TP53, while DNAH11 mutations conferred worse OS only • Gene-level gains/losses: 5 events conferring shorter OS, including losses of TP53/17p, CYLD/16q, FAT1, and amplifications of MYC and NRAS • Multivariate analysis for PFS: mutations in SP140 and NRAS, t(4;14), amp(1q), del(17p13) and deletions of FAT1 and PRDM1 • Multivariate analysis for OS: t(4;14), amp(1q), del(17p13), del(1p)
MMRF CoMMpass Study [19, 85]	1151 in total	Data from patients receiving treatment in the context of clinical trials as well as with real word regimens were included	• 55 genes were significantly mutated and there was a 65% overlap with the MGP • The linker histones HIST1H1B, HIST1H1D, HIST1H1E, and HIST1H2BK all showed a distinctive pattern of missense mutations clustered in the highly conserved globular domain • FUBP1, an important regulator of MYC transcription, showed an excess of splice site and nonsense mutations, emerging as a potential tumor suppressor gene in MM	• Multivariate analysis for early PD^c^ ◦ TP53 mutation (OR, 3.78, *P* < 0.001) ◦ High lactate dehydrogenase levels (OR, 3.15, *P*=0.006), ◦ IgL-chain translocation (OR, 2.25, *P* = 0.033) ◦ IGLL5 mutation (OR, 2.15, *P* = 0.007) • A trend was found for gain(1q) and amp(1q) in regards to early PD; but this did correlate with early death within 24 months • Survival analysis revealed significantly shorter PFS in patients with greater than average somatic missense mutation load (49.3 vs. 72.6% 2-year PFS, *P* = 0.0023) and predicted expressed neoantigen load (*N* = 214, 55.5 vs. 72.9% 2-year PFS, *P* = 0.0028)

*CA* cytogenetic abnormalities, *CCF* cancer clone fraction, *IMID* immunomodulatory drug, *LOH* loss of heterozygosity, *MGP* myeloma genome project, *MM* multiple myeloma, *MMRF* Multiple Myeloma Research Foundation, *OS* overall survival, *PD* progressive disease, *PFS* progression-free survival, *WES* whole-exome sequencing.

^a^Mutations in CRBN and IKZF1 have been associated with IMID resistance.

^b^Mutations in tumor suppressor genes co-occurred with deletion in the wild-type allele.

^c^Early progressive disease was defined as time to progression of less than 18 months.

## RNA and gene-expression profiling

Given DNA-based assays such as whole genomic sequencing are able to identify individual lesions and markers of global genomic instability and ultimately prognosis, it is not surprising that the development and now validation of several GEP scoring systems have shown strong prognostic value. Most studies have identified GEP signatures as an independent prognostic factor although overlap with clinical and iFISH/cytogenetic risk factors do exist [28–30]. The HOVON-65/GMMG-HD4 clinical trial researchers and University of Arkansas for Medical Sciences (UAMS) researchers have reported a 92 [30] and 70-gene signature [28], respectively, able to identify poor outcome in independent cohorts. Although a variety of other GEP have been developed [31], only two have matured into validated clinical tests: MMprofiler (EMC92/SYK92) and MyPRS (UAMS GEP70).

### EMC92/SKY92/MMprofiler

This GEP was originally developed from newly diagnosed MM patients included in the HOVON-65/GMMG-HD4 trial (*n* = 290) [30]. A prognostic signature of 92 genes (EMC92-gene signature) was generated with high-risk defined as OS of less than 2 years (63 out of 290 patients—21.7%) generating a two-tier system of high and standard-risk populations. The EMC92 was then validated in several up-front MM patient cohorts including total therapy (TT)2 (19.4% at high risk), TT3 (16.2% at high risk) and MRC-IX (20.2% at high risk). Multivariate analysis was performed in the training set and in the MRC-IX validation sets which showed that in addition to the EMC92 signature, del(17p) and β2M were also independent predictors in HOVON-65/GMMG-HD4. The SYK92 MMprofiler would go on to be validated in other in NDMM settings including patients receiving up-front KRD induction with and without ASCT consolidation [32]; 329 patients from the NRCI Myeloma XI trial [29]; and specifically in elderly non-transplant eligible patients [33].

### The UAMS GEP70 or MyPRS

In one of the earliest GEP studies, Shaughnessy et al reported on a 70-gene scoring system in 532 NDMM patients [28]. Both the training and validation groups were treated on National Institutes of Health (NIH)–sponsored clinical trials UARK 98–026 and UARK 03–033, respectively. Both protocols used chemotherapy-based induction regimens followed by melphalan-based tandem autotransplantation, consolidation chemotherapy, and maintenance treatment. They identified a high-risk group that comprised 13.4% of patients and exhibited significantly inferior event-free survival (EFS)(*P* = 0.001; HR of 4.51) and OS (*P* = 0.001; HR of 5.16). On multivariable analyses for OS and EFS controlling for ISS risk and high-risk translocations, the high-risk UAMS GEP70 score retained its significance (HR = 4.1; *P* = 0.001). As with the SYK92, this has now been validated in several cohorts including the same 329 NDMM patients treated on the NCRI Myeloma XI trial as well as 456 patients treated on the GMMG-MM5 trial [34].

### Is GEP ready for prime time?

Despite growing evidence of its prognostic value, the application to routine clinical care remains challenging. There is no consensus on a universal adaptation and none are validated by the FDA. Chng et al. attempted to evaluate the optimal GEP for MM by examining patients from three publically available GEP datasets [35]. They evaluated nine GEP profiles looking at all non-redundant combinations and constructed all possible combinations of multiple signatures up to nine full signatures and performed survival analysis for each combination. They demonstrated reproducibility across the nine systems, thus GEP can capture core biology that is not a result of random methodological artifact. They showed that the EMC92+HZDCD combination provides highly improved performance compared with other signatures or combinations. Others have shown that the SYK92 [36] or a combination of the EMC92 and the ISS (referred to as the EMC92-ISS) may be the optimal system [37]. With a rapidly changing therapeutic landscape, re-validation will be necessary. Capturing clonal content and evolution remains a challenge and newer high-throughput technologies are needed along with newer bioinformatics methodologies to identify meaning from the large amount of data being generated. Many unanswered questions still exist such as different GEP mutual relationships, the utilization of multiple systems, and the possibility of outperforming combinations. Nevertheless, targeted NGS approaches allow the assessment of all copy number variations, IGH translocations, and recurrent mutations in one technique. Thus, likely this technology has significant advantages in the long term [35–37].

## Beyond the R-ISS: high-risk clinical features

**Fig. 1:**
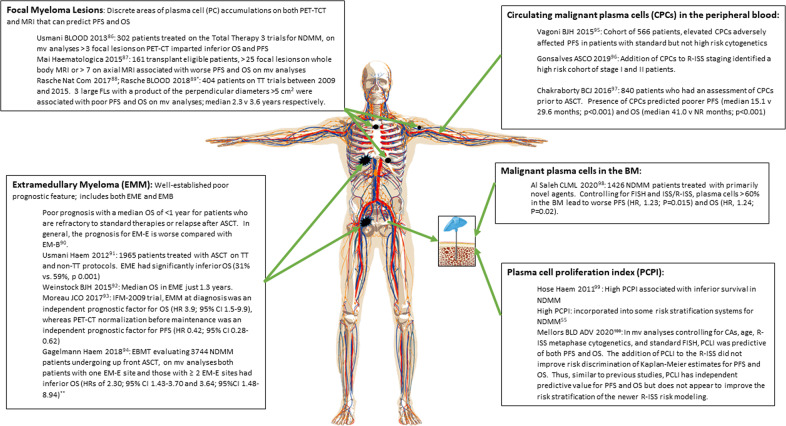
High-risk clinical features. ^*^Large FLs (diameter >2.5 cm) associated with site-specific enrichment of HiR driver mutations consistent with them being key mediators of drug resistance and treatment failure [86–100]. ^**^Certain EME sites seemed to carry worse prognosis with 3-year PFS differing according to involved organs: kidney (59.5%), skin (20.1%), lymph nodes (37.6%), CNS (47.9%), lung/respiratory tract (44.4%), GI/liver (22.5%), and spleen, ovaries, and testes (60.0%). BM bone marrow, CA cytogenetic abnormalities, CPCs circulating plasma cells, EBMT European Society for Blood and Marrow Transplantation, EME extramedullary myeloma that is extra-osseous (results from hematogenous spread and involving only soft tissues, the incidence in NDMM 1.7–3.5%^90^), EMB extamedullary myeloma that is paraskeletal or paraosseous plasmacytomas (consists of tumor masses adjacent to bones and arising from focal skeletal lesions, incidence in NDMM 6–34.4%^90^), EMM extramedullary myeloma, FL focal lesion, HR hazard ratio, ISS international staging system, MRI magnetic resonance imaging, MV multivariate, NDMM newly diagnoses multiple myeloma, NR not reached, OS overall survival, PC plasma cells, PCPI plasma cell proliferation index, PET-CT 18-fluoro-deoxyglucose emission tomography, PFS progression-free survival, R-ISS revised international staging system, TT total therapy.

Clinical and biological features have prognostic value beyond genomics in NDMM patients. Tumor burden dictates risk and was included in the original ISS staging system [3]. Subsequently, malignant plasma cells in the bone marrow and peripheral blood have also been shown to be prognostic. The plasma cell proliferation index (PCPI), a measure of plasma cell proliferative activity, has shown an association between metaphase cytogenetic abnormalities and rapid myeloma cell proliferation and ultimately clinical outcomes [38]. Focal myeloma lesions and extramedullary disease have also been shown to predict clinical outcomes. However, questions remain regarding the potential confounding of genomics on these high-risk biological and disease burden-related risk factors. Disease burden and patient-related factors depicting risk are outlined in Fig. 1.

### Patient-related factors

In addition to risk-stratification systems, genomic features, and disease burden, additional non-modifiable patient-related factors affect outcomes in MM. Clinical frailty and geriatric assessments have been shown to impact outcomes in MM but their routine use has been largely limited due to clinical time restraints. In a recent systemic review and meta-analysis, a significantly increased HR for death was shown for patients with activity of daily living score ≤4 (pooled HR = 1.576; 95% CI, 1.051–2.102) [39]. Further, patients classified as frail showed higher risk of death than fit patients did (pooled HR = 2.169; 95% CI, 1.002–2.336). It is of note though that genomic risk may be intimately related with patient-related factors. In 1777 NDMM patients treated on the Myeloma XI trial, patients with TP53 deletion showed features of advanced disease and associated morbidity, specifically poorer performance status (World Health Organization [WHO] performance status ≥2; *P* = 0.0012). Although WHO performance status was independently associated with shorter survival, the association with TP53 deletion suggests an interrelationship with genetic and clinical features [40].

There is increasing evidence that socioeconomics and access to care directly impact patient outcomes. Several studies have demonstrated that patients of minority ethnic or racial background are less likely than non-Hispanic Whites (nHws) to receive ASCT as treatment for MM and that referral for transplantation may be delayed. However, similar outcomes for minorities compared with nHws undergoing ASCT has been shown when access is equal [41]. MM patients of racial and ethnic minority are frequently underrepresented in clinical trials. Pulte et al. performed a meta-analysis evaluating patients on five recent clinical trials that utilized novel agents and did not find a difference in outcome based on race. Because Hispanic and African American patients have the least apparent benefit from newer agents at the population level. These results suggest that minority patients are less likely to be appropriately treated [42]. To further validate this point, a recent VA experience showed that with equal access, AA patients may have superior outcomes with median OS of AA patients 5.07 years (95% CI, 4.70–5.44 years) as opposed to 4.52 years (95% CI, 4.38–4.65 years) for white veterans (log-rank *P* < 0.001) [43].

### Biology of disease trumps everything

Response to initial therapy and achieving a prolonged initial remission duration may ultimately be the most important prognostic factor in NDMM patients. There is clear data that shows achieving deep remissions that are minimal residual disease (MRD) negative can trump high-risk biological features and that standard-risk patients who fail to achieve deep remissions fair worse and may indeed be high risk after all [44]. Below, we will briefly review the data on primary refractory and early relapsing myeloma but will forgo an in-depth review of MRD and its impact on outcomes as this topic has been covered extensively in several recent reviews and meta-analyses.

Response rates to standard triplet induction therapy for both transplant eligible and ineligible patients are in the 85–90% range [45] thus primary refractory myeloma is uncommon. Unfortunately, despite improved 2nd line therapy, outcomes for these patients remain poor even if treated with novel induction. For patients undergoing up-front ASCT after induction failure, as far back as 2010 Gertz et al. showed that failure to achieve at least a partial response (PR) to IMID based induction prior to ASCT leads to shorter OS (73.5 vs. 30.4 months) and PFS (22.1 vs. 13.1 months; *P* < 0.001) from time of transplant [46]. Lee et al. demonstrated even worse outcomes in patients refractory to novel based regimens (majority were bortezomib based) showing a median PFS of 4.7 months and median OS of 11.6 months following ASCT [47]. Although there is limited data in transplant ineligible or deferred patients, the same pattern holds. For example, in an updated analysis from the mayo clinic amongst patients treated with novel induction regimens, primary refractory patients had a far inferior median OS of just 3.6 vs. 7.9 years (*P* < 0.001) [48].

Early relapse is likely a reflection of the underlying high-risk disease biology that was not captured in the initial risk assessment and leads to inferior outcomes regardless of cytogenetic risk. Durie et al. were the first to show that the underlying dominant predictor for survival is time to progression [49] and the Mayo Clinic was the first to describe the adverse prognostic impact of an early relapse after intensive strategy [50]. In a Center for International Blood and Marrow Transplant Research (CIBMTR) analyses of 3256 NDMM patients from 2001 to 2013 who received up-front ASCT, the proportion of patients relapsing within 24 months following ASCT was stable over time at 35–38%. The OS from the time of relapse was significantly inferior for the early relapse group with a 4-year OS of 30% vs. 41% (*P*<0.001) [51]. Relapse within 1 year of ASCT leads to even worse outcome with Kastritis et al. showing that among 297 consecutive NDMM patients receiving first-line ASCT, 43(14.5%) relapsed within 12 months and had dismal outcomes with median post-ASCT survival of 18 months vs. >6 years (*P*<0.001) in late relapsing patients [51]. These outcomes unfortunately have not improved much with an older cohort from the Mayo clinic showing just a 23.9-month median OS [52].

Patients not eligible for up-front ASCT who relapse early also do poorly. In a cohort of 511 NDMM patients, Majithia et al. showed that in 82 patients (16%) who relapsed within one year of therapy, the median OS was 21.0 months vs. NR (*P*<0.001). The survival disadvantage persisted even when considering only patients who received subsequent therapies with a median OS of 26.7 months vs. NR (*P*<0.001) [53]. Finally, a recent IFM report showed that early relapse after first-line therapy still negatively impacts survival even when controlled for genomic factors [7]. Interestingly, approximately two-thirds of early relapsing patients in this IFM cohort were not initially considered high risk and thus early relapse trumps genomic risk.

## Conclusion: developing a new high-risk model and future directions

The myeloma research community has amassed a vast expanse of genomic data from NDMM patients over the last decade. This has led to significant advances in our understanding of the genomic changes that portend to poor outcomes in NDMM patients. Unfortunately, our success in elucidating high-risk genomic features in NDMM patients has not translated into tailored therapeutics and improved outcomes in these patients. An up-to-date uniform consensus on high-risk features is overdue and expected soon from the IMWG. Table 5 outlines our current stance on high-risk features in NDMM patients. Certain features, such as GEP, whole-genome sequencing, and PCLI may not be applicable in routine clinical practice but nonetheless have been consistently shown to drive poor outcomes. More comprehensive and routinely obtained genomic profiling beyond traditional FISH is needed to advance risk stratification in NDMM. We would consider any NDMM patient that meets any of the criteria listed in the high-risk column as being a high-risk patient and strongly encourage enrollment onto clinical trials for these patients.

**Table 5:** High-risk features for newly diagnosed multiple myeloma.

	High risk	Potentially high risk (more data needed)
Currently Utilized Staging systems:	R-ISS stage 3 IMWG high-risk mSMART high risk	
High-risk cytogenetic changes^a^	• t(14;16) • t(4;14) • IgL-MYC translocation • +1q amplification (≥4 copies): 20% CCF • 1p- • del(17p): 55–60% CCF	• t(14;20) • t(8;14) and other MYC translocations • +1q gain (3 copies) • del 13q/-13
GEP-results	EMC92/SYK92 (MMprofiler): high-risk UAMS GEP70 (MyPRS): high risk	
Mutations obtained by whole-genome/exome sequencing	• TP53 deletion • LOH and APOEBEC signature • CKS1B amplification • "High Risk Genomic Clusters"^b^	• TRAF3 • TGDS • PRDM1 • DNAH11 • FAT1 • NRAS • SP140 • IGLL5 • Driver gene mutational burden
Clinical Features and disease burden:	• High Plasma Cell Labeling Index • Extramedullary Myeloma • Focal Lesions (FL): 3 large FLs with a product of the perpendicular diameters >5 cm^2^ • Clinical frailty by objective geriatric assessment	Socioeconomic status

*GEP* gene-expression profiling, *IMWG* international myeloma working group, *LOS* loss of heterozygosity, *MM* multiple myeloma, *R-ISS* revised international staging system.

^a^Translocations and copy number abnormalities (independent of other features) with cancer clone fraction cutoffs where enough data supports a conclusion.

^b^See Table 4.

In order to properly risk-stratify patients in routine clinical care, we recommend obtaining the following at diagnosis prior to initiating therapy:

- Serum studies: LDH, β_2_-microglobulin, albumin
- Imaging: skeletal survey, advanced bone imaging ideally PET-CT (alternatively whole-body CT, MRI spine and pelvis)
- Bone marrow biopsy: standard cytogenetics, iFISH myeloma panel, clonoseq MRD ID specimen, GEP, and PCPI as able
- Frailty/performance status and socioeconomic barriers to care.

MM is a genomically complex disease with diverse clinical outcomes based on the genomic footprint of each individual patient. The international collaboration of MM practitioners has advanced both our biological understanding of risk in myeloma and has led to improved treatment outcomes overall. Moving forward, several challenges remain and ongoing large-scale collaboration will be needed to overcome them. We must begin a more concerted effort to translate our knowledge of high-risk genomic features into improved clinical outcomes by tailoring therapeutics to risk. The standardization of iFISH methodology and importantly the definition of positive results is needed. We must move to incorporate GEP and possibly PCLI into routine clinical care not just at large academic centers and as part of clinical trials. We must better incorporate objective measurements of patient-related factors into our risk assessment and treatment approach. Finally, we must address access to myeloma care to overcome socioeconomic barriers to care that have led to inferior outcomes in ethnic minorities diagnosed with MM. These challenges are immense but with ongoing collaboration, they can be achieved in time.

## Data Availability

This article file has no independent data.
